# Pharmacokinetic Properties of Fluorescently Labelled Hydroxypropyl-Beta-Cyclodextrin

**DOI:** 10.3390/biom9100509

**Published:** 2019-09-20

**Authors:** Judit Váradi, Anca Hermenean, Rudolf Gesztelyi, Viktória Jeney, Enikő Balogh, László Majoros, Milo Malanga, Éva Fenyvesi, Lajos Szente, Ildikó Bácskay, Miklós Vecsernyés, Pálma Fehér, Zoltán Ujhelyi, Gábor Vasvári, István Árvai, Ágnes Rusznyák, Cornel Balta, Hildegard Herman, Ferenc Fenyvesi

**Affiliations:** 1 Department of Pharmaceutical Technology, Faculty of Pharmacy, University of Debrecen, Nagyerdei St. 98, H-4032 Debrecen, Hungary; 2 Department of Histology, Faculty of Medicine, ‘Vasile Goldiș’ Western University of Arad, 86 Rebreanu Street, 310414 Arad, Romania; 3 Department of Biochemistry and Molecular Biology, University of Bucharest, Splaiul Independenței Street, no. 91–95, sector 5, 050095 Bucharest, Romania; 4 Department of Pharmacology and Pharmacotherapy, Faculty of Medicine, University of Debrecen, Nagyerdei St. 98, H-4032 Debrecen, Hungary; 5 MTA-DE Lendület Vascular Pathophysiology Research Group, Research Centre for Molecular Medicine, Faculty of Medicine, University of Debrecen, 4012 Debrecen, Hungary; 6 Department of Medical Microbiology, Faculty of Medicine, University of Debrecen, Nagyerdei St. 98, H-4032 Debrecen, Hungary; 7 Cyclolab Cyclodextrin R&D Laboratory Ltd., Illatos St. 7, H-1097 Budapest, Hungary; 8 University of Debrecen, Doctoral School of Pharmaceutical Sciences, Nagyerdei St. 98, H-4032 Debrecen, Hungary

**Keywords:** hydroxypropyl-beta-cyclodextrin, pharmacokinetics, HUVECs, endocytosis, fluorescence

## Abstract

2-Hydroxypropyl-beta-cyclodextrin (HPBCD) is utilized in the formulation of pharmaceutical products and recently orphan designation was granted for the treatment of Niemann–Pick disease, type C. The exact mechanism of HPBCD action and side effects are not completely explained. We used fluorescently labelled hydroxypropyl-beta-cyclodextrin (FITC-HPBCD) to study its pharmacokinetic parameters in mice and compare with native HPBCD data. We found that FITC-HPBCD has fast distribution and elimination, similar to HPBCD. Interestingly animals could be divided into two groups, where the pharmacokinetic parameters followed or did not follow the two-compartment, first-order kinetic model. Tissue distribution studies revealed, that a significant amount of FITC-HPBCD could be detected in kidneys after 60 min treatment, due to its renal excretion. Ex vivo fluorescent imaging showed that fluorescence could be measured in lung, liver, brain and spleen after 30 min of treatment. To model the interaction and cellular distribution of FITC-HPBCD in the wall of blood vessels, we treated human umbilical vein endothelial cells (HUVECs) with FITC-HPBCD and demonstrated for the first time that this compound could be detected in the cytoplasm in small vesicles after 30 min of treatment. FITC-HPBCD has similar pharmacokinetic to HPBCD and can provide new information to the detailed mechanism of action of HPBCD.

## 1. Introduction

2-Hydroxypropyl-beta-cyclodextrin (HPBCD) is widely utilized as pharmaceutical excipient in several products. On the other hand, orphan designation (EU/3/11/895) was granted by the European Commission for hydroxy-propyl-beta-cyclodextrin for the treatment of Niemann–Pick disease, type C (NPC) in 2011 [1]. Between 2013 and 2015, a non-randomized, open-label, phase 1–2 trial was conducted to establish the safety and efficacy of lumbar intrathecal HPBCD in patients with NPC1 [2]. The result of the study showed, that HPBCD slowed disease progression with an acceptable safety profile. Indeed, HPBCD is listed in the Food and Drug Administration’s (FDA) list of inactive pharmaceutical ingredients [3] and a previous publication showed, that intravenously (i.v.) administration of HPBCD is safe and well tolerated [4,5]. HPBCD is always a mixture of isomers with various degrees of substitution (DS), which may influence the complexation ability of the cyclodextrins [6]. We also tested the cytotoxicity and hemolytic activity of HPBCD with different DS and found no toxicity on Caco-2 cells and low hemolytic activity [6,7]. Pharmacokinetic studies of i.v. HPBCD revealed fast elimination and distribution over the extracellular fluid. I.v. HPBCD is eliminated by glomerular filtration from the blood and had only reversible vacuolation in kidneys but did not cause kidney dysfunction in animals or humans [4,5]. However, earlier studies reported that subcutaneous long-term administration of HPBCD at a daily dose of 200 mg/kg caused bone loss in rats [8], 2 week 0.25–0.5 g/kg/infusion of HPBCD caused inflammatory changes in the lung of treated pigs [9] and foamy macrophage infiltration of the lung after 4–7 days 225 mg/kg/day i.v. in rats [4]. Ototoxicity is another reported risk [10], which was found in cats [11] mice [12,13] and in humans [2], but the mechanism of ototoxicity is unknown [10]. It was also revealed using fluorescent cyclodextrin derivatives, that HPBCD (and other cyclodextrins) is able to enter the cytoplasm of different cell types by endocytosis [14,15,16,17,18]. This process can be essential for the effects of HPBCD. The pharmacokinetic of native HPBCD after different routes of administration was characterized in animal models and in humans [19,20], but to discover the exact mechanism of action at the level of tissues and cells, sensitive methods are required. Radiolabeling of cyclodextrins [21] and covalent conjugation of fluorophores to the cyclodextrin ring are possible tools for detection [22]. Fluorescent HPBCD derivatives can be applied to study the behavior of HPBCD at the level of tissues and cells and may help to understand its effects and side effects. We aimed to reveal the pharmacokinetic parameters and tissue distribution of FITC-HPBCD in mice, administered intravenously and compare the data of HPBCD available in the literature. We also investigated the interaction of FITC-HPBCD with human endothelial cells, which constitute the first barrier in the body after i.v. administration of drugs.

## 2. Materials and Methods

### 2.1. Materials

6-deoxy-6-[(5/6)-fluoresceinylthioureido]-(2-hydroxypropyl)-β-cyclodextrin (FITC-HPBCD) was synthesized on the analogy of rhodaminyl HPBCD as described earlier [23]. FITC-HPBCD was the product of Cyclolab Ltd. (Budapest, Hungary). FITC-HPBCD product number: CY-F-2005.1, average degree of substitution (DS) determined by NMR were DS = 0.7 for FITC and DS = 4.1 for hydroxypropyl groups. FITC-HPBCD was purified by extensive dialization to remove impurities. Free dye and 6-deoxy-6-monoamino-HPBCD contents were tested by capillary electrophoresis (CE) and were below the limit of detection. All other reagents were from Sigma-Aldrich (Budapest, Hungary).

### 2.2. Cell Culture

Human umbilical vein endothelial cells (HUVECs) were removed from umbilical veins by exposure to dispase (0.2% in Hank’s balanced salt solution) and cultured in gelatin-coated flasks in medium 199 containing 15% foetal bovine serum (FBS), antibiotics, L-glutamine, sodium pyruvate and endothelial cell growth supplement from bovine pituitary [24]. For the fluorescent microscopic experiments, cells were seeded onto gelatin-coated round glass coverslips and maintained in medium until the treatments.

### 2.3. Intracellular FITC-HPBCD Accumulation in HUVEC Cells

#### 2.3.1. Fluorescence Microscopy

HUVECs, cultured on gelatin-coated glass coverslips were washed twice with Hank’s Balanced Salt Solution (HBSS) and treated with 50 µM FITC-HPBCD for 30 min at 37 °C. Cells were washed five times with ice-cold HBSS and fixed with 3% paraformaldehyde for 15 min. After fixation cells were washed with HBSS and the nuclei were stained with Hoechst 33,342 at the final concentration of 300 nM. Cells were washed again and mounted on microscope slides. Samples were observed with a Zeiss Axio Scope.A1 fluorescence microscope (HBO 100 lamp) (Carl Zeiss Microimaging GmbH, Göttingen, Germany). Images were analyzed with ZEN 2012 v.1.1.0.0. software (Carl Zeiss Microscopy GmbH, Göttingen, Germany)

#### 2.3.2. Flow Cytometry

In the flow cytometry experiments, HUVECs were treated with 50 µM FITC-HPBCD for 30 min at 37 °C and washed five times with ice-cold HBSS. Half of the samples were incubated for a further 60 min in HBSS at 37 °C. After the above-mentioned incubations every sample was trypsinized, the cell suspensions were washed twice with ice-cold HBSS and analyzed with a FACScan flow cytometer (BD Biosciences, San Jose, CA, USA). Propidium-iodide (PI) (2 µg/mL) was added to the samples to recognize dead cells and exclude them from the evaluation. FITC-HPBCD was excited by an argon laser at 488 nm and the emission was detected via 530/30 nm band pass filter. Data were analyzed by BDIS Cellquest software (Becton-Dickinson). The cellular fluorescence intensities of the FITC-HPBCD treated cells were compared to the fluorescence of the untreated control cells and expressed as relative cellular fluorescence intensities.

### 2.4. In Vivo Pharmacokinetic Investigation

Twelve BALB/c male mice (23–25 g) were involved in the study. 0.25 mg FITC-HPBCD, dissolved in 0.2 mL saline solution was injected into the lateral tail vein of each mouse. Blood samples were collected from the orbital sinus from six mice after 1, 15, 30 and 60 min and at 1, 15, 45 and 60 min from other six mice by the same sampling method. Blood samples were incubated for 1 h at 37 °C and centrifuged for 8 min at 1700 rpm to separate blood plasma. Aliquots of plasma from the samples were placed into black 96-well plates and their fluorescence intensities were measured by a FLUOstar Optima microplate reader (BMG LABTECH, Offenburg, Germany; 492 nm excitation and 520 nm emission wavelengths) and the plasma FITC-HPBCD concentrations were calculated. Using these data, the main pharmacokinetic parameters were determined: rate constants of elimination (k_e_) and compartmentalization (k_c_), volume of distribution (V_d_), clearance (CL) and half-life (t_1/2_):

(1) $$t_{1/2}=\frac{\ln\left(2\right)}{k_{e}}$$

CL = k_e_ × V_d_ (2)

The two compartment model was characterized by the following equations:

(3) $$C_{t}=C^{\prime }\times e^{-k_{c}\times t}+C^{\prime \prime }\times e^{-k_{e}\times t}$$

(4) $$C_{0}=C^{\prime }+C^{\prime \prime }$$

where k_e_ is the elimination rate constant of FITC-HPBCD determined from data of the terminal phase (k_e(30-60 min)_ or k_e(45-60 min)_); t_1/2_ is the half-life of FITC-HPBCD calculated from the corresponding k_e_ values; CL is the clearance of FITC-HPBCD; V_d_ is the volume of distribution of FITC-HPBCD determined from data related to the first minute (V_d1_); C_t_ is the plasma concentration of FITC-HPBCD at “t”; C′ and C″ are the two components of the initial plasma concentration of FITC-HPBCD representing the amount of FITC-HPBCD that will be compartmentalized and eliminated, respectively.

After the last blood sample collection, six animals were anaesthetized, and their blood vessels were perfused by warm saline solution. Finally, all mice were sacrificed, and the organs were removed from the six saline-perfused mice for further analysis. The collected organs (livers, lungs, kidneys, spleens, hearts and brains) were homogenized by adding 0.8 mL saline solution and the homogenates were centrifuged at 10,000 rpm for 20 min at 4 °C. From each sample, 0.1 mL of supernatant was placed into black 96-well plates and the fluorescence intensities were measured by microplate reader as described above. The lower limit of quantification of FITC-HPBCD in mouse plasma and organ homogenates was 0.25 µg/mL. The protein content of the tissue samples was determined by PierceTM BCA protein assay (Thermo Scientific, Rockford, IL, USA) according to the manufacturer’s instruction. FITC-HPBCD content of the samples was normalized to the tissue protein content. The animals were maintained in accordance with the Guidelines for the Care and Use of Laboratory Animals; experiments were approved by the Animal Care Committee of the University of Debrecen (permission no. 12/2014).

### 2.5. In Vivo Fluorescence Imaging Experiments

Male BALB/c mice (22–25 g) were bred and maintained in normal watering and feeding conditions in a Laboratory Animal Husbandry Facility, equipped with IVC (individually ventilated cages), controlled atmosphere-temperature, humidity and lighting (Vasile Goldis Western University, Arad, Romania). All experimental procedures were performed in compliance with the appropriate laws and institutional guidelines, and were approved by the Institutional Ethics Committee of Vasile Goldiș Western University of Arad (Arad, Romania). FITC-HPBCD (0.5 and 2.5 mg), dissolved in 0.1 mL saline solution was injected into the femoral vein of mice under general anaesthesia. Maintaining the surgical plane of anesthesia, mice were placed into an in vivo imaging system (In-vivo Xtreme, Carestream, Carestream Health Inc. USA). At 5, 15, 30, 45 and 60 min, fluorescence and X-ray images were recorded of the animals. After 30 or 60 min, the blood vessels of some animals were perfused by warm saline solution and their organs (livers, lungs, kidneys, spleens and brains) were removed for ex vivo imaging (excitation: 480 nm, emission: 535 nm).

### 2.6. Statistical Analysis

SigmaStat software (version 3.1, SPSS Inc.) was used for statistical analysis. Data are expressed as means ± SD. Comparison of groups was perfomed by ANOVA. Differences were considered significant at *p* < 0.05.

## 3. Results

### 3.1. In Vivo Pharmacokinetic Investigation

**Figure 1:**
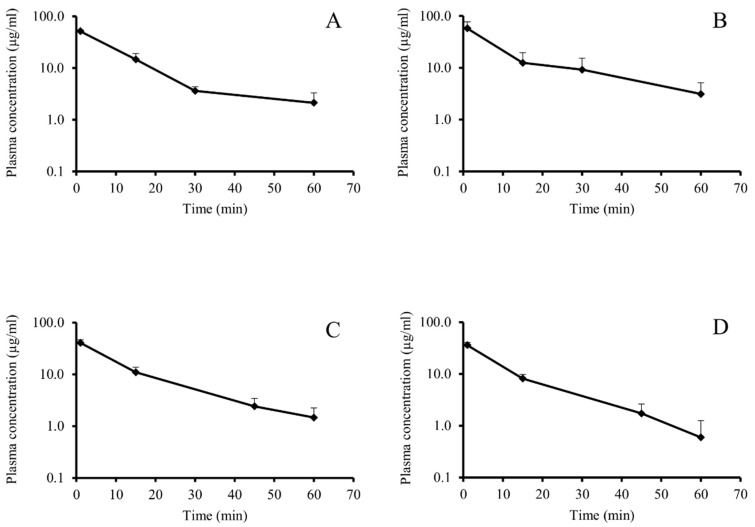
Plasma concentration curves of FITC-HPBCD after i.v. administration in mice. Sampling times 1, 15, 30 and 60 min (**A** and **B**; *n* = 6) and 1, 15, 45 and 60 min (**C** and **D**; *n* = 6). A and C followed the first-order elimination kinetic, while B and D did not follow it.

At first, our aim was to find the best model for the pharmacokinetics of FITC-HPBCD and determine its pharmacokinetic parameters. We applied two experimental protocols. In the first case, we took blood samples from the animals at 1, 15, 30 and 60 min, and in the second case, at 1, 15, 45 and 60 min. When data of FITC-HPBCD were evaluated, the two-compartment model with the assumption of first-order elimination kinetics showed the best fit. During the calculation of the rate constants of elimination (k_e_) and compartmentalization (k_c_), we identified two distinct groups of animals. In the first group (Group F), a k_c_ value greater than k_e_ could be computed (so, the behavior of FITC-HPBCD in the animals of Group F was consistent with expectations resulted from the two-compartment, first-order kinetic model) (Table 1 and Figure 1A,C). However, in the second group (Group NF), k_c_ could not be determined (thus, the fate of FITC-HPBCD did not follow expectations of our model) (Table 1 and Figure 1B,D). Interestingly, both groups contained six mice, irrespectively of the sampling protocol used.

FITC-HPBCD showed fast elimination in mice after i.v. administration. Within 60 min 96.58 ± 2.6% (*n* = 12) of the i.v. administered FITC-HPBCD was eliminated from mice. Despite the fast elimination, FITC-HPBCD showed rapid compartmentalization (confirmed by the calculation of volume of distribution: V_d_). V_d_ was higher than the blood volume of mice, at the first minute, it was 5.7 ± 1.34 mL (*n* = 12). There was no significant difference between V_d_ values of the two groups with different elimination parameters. Compartmentalization and elimination of the compound in question happened at the same time in the animals, but in the group not following the kinetic model, distribution appeared to be too complex for a simple interpretation.

**Figure 2:**
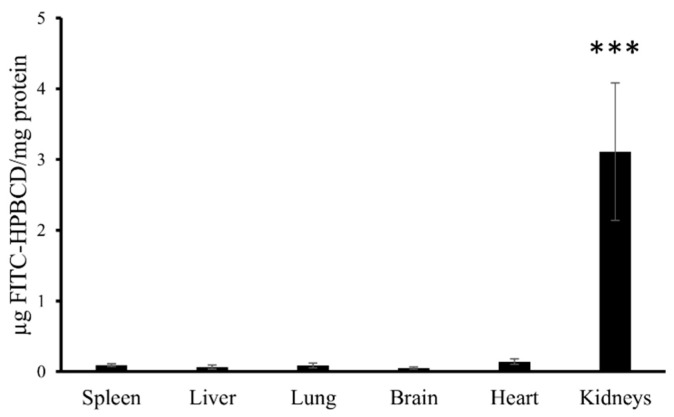
FITC-HPBCD content in the supernatant of the tissue homogenates of mice. FITC-HPBCD was administered i.v. and after 60 min of treatment significant fluorescence intensity could be measured only in the supernatant of kidney homogenates (*** *p* < 0.001).

After 60 min of the pharmacokinetic experiment, we analyzed the tissue accumulation of FITC-HPBCD in tissue homogenates ex vivo. The fluorescence intensities of FITC-HPBCD in the supernatant of liver, lung, spleen, heart and brain homogenates were not elevated after 60 min of the experiment. Only the homogenates of kidneys showed significant fluorescence (Figure 2).

### 3.2. In Vivo Fluorescence Imaging Experiments

**Figure 3:**
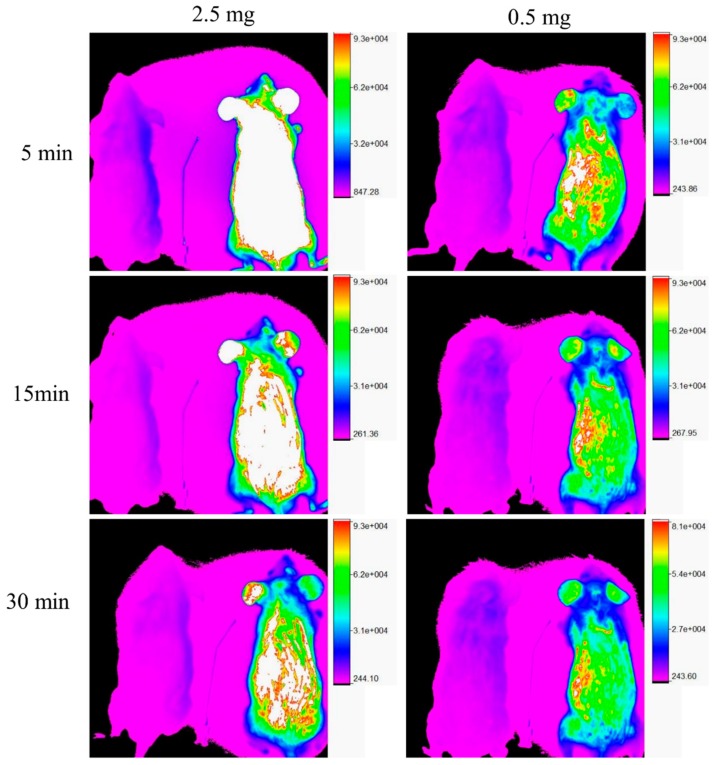
Representative in vivo fluorescence images of FITC-HPBCD treated mice. FITC-HPBCD (0.5 and 2.5 mg) was injected i.v. and images were recorded at 5, 15 and 30 min after injection. Untreated mice can be seen on the left and FITC-HPBCD treated mice can be seen on the right in each image. FITC-HPBCD distributed over the entire body of mice and the in vivo fluorescence decreased as a function of time.

Two doses (0.5 mg and 2.5 mg/animal) of FITC-HPBCD were administered to the mice i.v. in the in vivo fluorescence imaging experiments. Images were recorded at 5, 15, 30, 45 and 60 min. In both cases, significant fluorescence could be observed in the animals (Figure 3, mice on the right in the images), compared to the control mice (Figure 3, mice on the left in the images). The compound was distributed over the entire body of the animals and their fluorescence intensities decreased as a function of time. 

**Figure 4:**
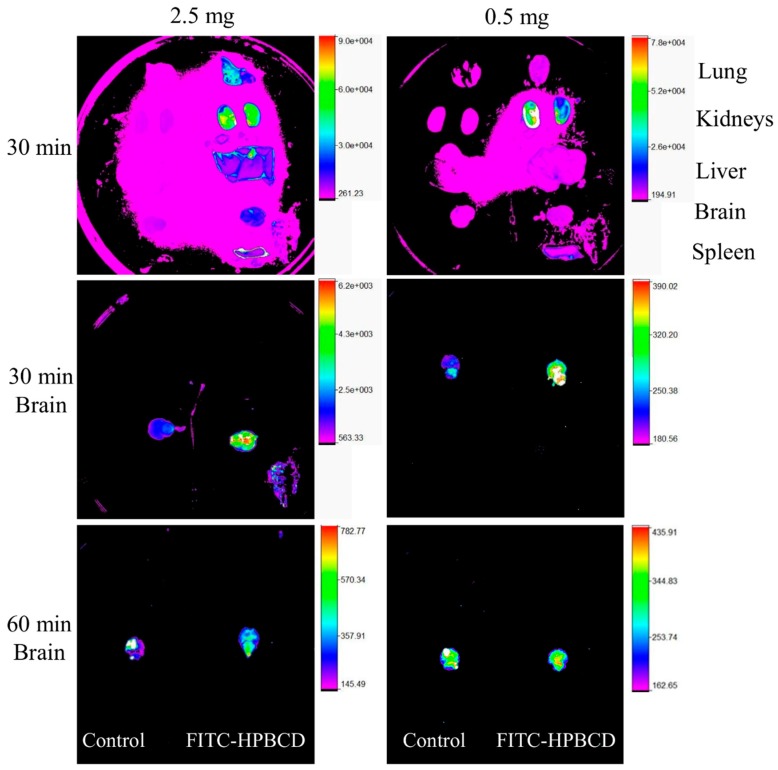
Representative ex vivo fluorescence images of the organs of FITC-HPBCD treated mice after 30 min treatment. Brains were examined separately from other organs after 30 and 60 min of treatment and imaged from beneath. The organs of untreated mice can be seen on the left in the images. Kidneys showed the highest fluorescence intensities in both doses (0.5 and 2.5 mg). In the case of 2.5 mg dose, lung also had elevated intensity. At 30 min the brains of the FITC-HPBCD treated animals had higher fluorescence than the controls, while after 60 min there was no difference between the fluorescence of the brains.

After 30 or 60 min, animals were perfused with warm saline solution and their organs were removed to measure the ex vivo fluorescence intensities. In case of the 2.5 mg/animal dose, every organ showed higher fluorescence intensities (Figure 4, organs on the right) than the control samples (Figure 4, organs on the left). Kidneys had especially high fluorescence and lung showed elevated intensity. From the administration of 0.5 mg FITC-HPBCD, the fluorescence intensities of the removed organs were similar to the control when imaged together, only kidneys had relative higher fluorescence after 30 min. Separated examination of brains with different gains revealed that in both doses, the brains of the treated animals had higher fluorescence than the untreated ones after 30 min. After 60 min there was no difference between the organelle fluorescence of the brains, the intensity of the treated sample decreased to the control level. 

Interestingly, lungs also showed higher ex vivo fluorescence in separated imaging after 0.5 mg FITC-HPBCD administration compared to the untreated control (see Supplementary Material Figure S1). It could be observed that FITC-HPBCD accumulated in the bladder (30 and 45 min) and then was urinated out (60 min) (see Supplementary Material Figure S2).

### 3.3. Intracellular FITC-HPBCD Accumulation in HUVEC Cells

#### 3.3.1. Fluorescence Microscopy

**Figure 5:**
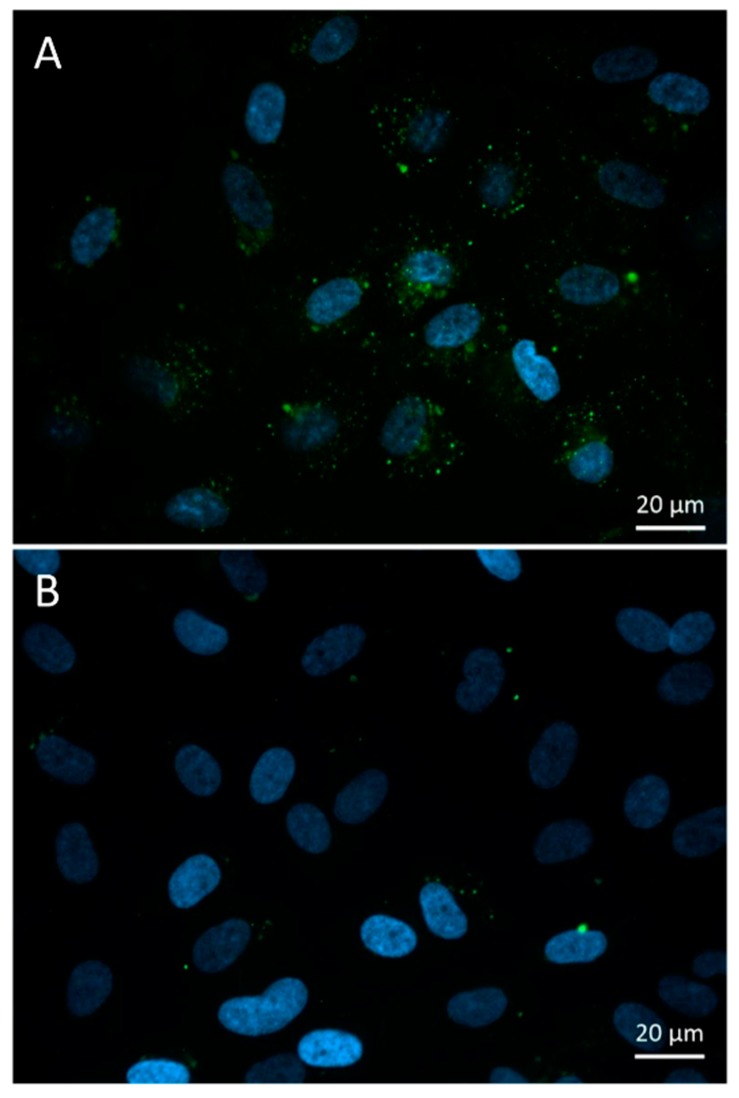
Fluorescence microscopic images of FITC-HPBCD treated (**A**) and untreated (**B**) human umbilical vein endothelial cells (HUVECs). Endocytotic green vesicles can be observed in the cytoplasm (**A**), while some autofluorescent vesicles can be seen in the control cells (**B**). (Green—FITC-HPBCD, blue—cell nuclei).

FITC-HPBCD could be detected in the cytoplasm of HUVECs in small green vesicles after 30 min of incubation. Some larger, more intense vesicles could be observed around the cell nuclei (Figure 5). In control cells, some autofluorescent vesicles could be identified.

#### 3.3.2. Flow Cytometry

**Figure 6:**
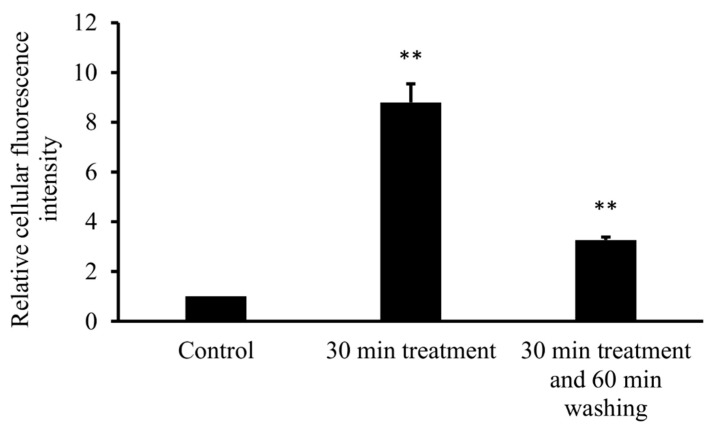
Flow cytometric analysis of FITC-HPBCD uptake in HUVECs. Incubation of HUVECs for 30 min with 50 µM of FITC-HPBCD significantly increased the cellular fluorescence and a following 60 min washing with HBSS decreased it. (Data are expressed as means ±SD, *n* = 3, significance is expressed as **p < 0.01).

To confirm cellular uptake of FITC-HPBCD by HUVECs, flow cytometric measurements were performed. After 30 min of incubation with FITC-HPBCD and extensive washing of cells, the cellular fluorescence was quantified by flow cytometer. FITC-HPBCD treatment significantly increased the cellular fluorescence, compared to the untreated control. Incubation of cells for 60 min in HBSS after 30 min of FITC-HPBCD treatment significantly decreased the fluorescence of HUVECs (Figure 6).

## 4. Discussion

In recent years, much attention was paid to the pharmacological and toxicological effects of cyclodextrins, especially to HPBCD. HPBCD is widely used excipient in oral and parenteral formulations, furthermore it is applied as orphan drug in the treatment of Niemann–Pick disease, type C. The pharmacokinetic properties of HPBCD was studied in rats [19] and humans [2,5,25], but the tissue distribution and the interactions with organs of this compound was moderately revealed. Mainly the nephrotoxicity was considered. It is known that HPBCD has extracellular distribution and fast elimination by glomelural filtration. FITC-HPBCD provides an excellent tool to study the above-mentioned questions and the molecule can also be detected sensitively at a cellular level. Moreover, FITC-HPBCD was successfully used to monitor renal functions [26]. In our experiments, FITC-HPBCD was administered to mice i.v. and blood samples were collected according to two protocols. At first, blood samples were collected at 1, 15, 30 and 60 min from six mice. Then the experiments were repeated by applying 1, 15, 45 and 60 min sampling times with six other animals and the pharmacokinetic parameters of mice varied in a relatively narrow range (Table 1). The t_1/2_ and k_e_ values calculated from data belonging to different time periods were different, thus they showed, that FITC-HPBCD had no simple elimination. As FITC-HPBCD was administered i.v., the most likely reason of the variation of k_e_ values was the presence of more than one compartment for FITC-HPBCD. k_e_ values decreasing over time are well fitted to the characteristics of a two-compartment model. Among the presented k_e_ values, the k_e_ belonging to the terminal phase (k_e (30–60 min)_ or k_e (45–60 min)_) was considered to be closest to the truth. Thus, this k_e_ value was used for the calculation of CL, as CL also characterizes the elimination. On the other hand, FITC-HPBCD was eliminated rapidly into the urine, and V_d_ was the least biased by elimination at the first minute (V_d1_), because at the beginning of the process compartmentalization was more significant. As a result, k_e (30–60 min)_ or k_e (45–60 min)_ and V_d1_ were used for the calculation of CL. k_c_ was also calculated from data at the first time period (k_c (1–15 min)_).

The data evaluation revealed that the animals could be divided into two groups, where the pharmacokinetic parameters followed or did not follow the two-compartment model. Interestingly, the number of the animals in the group following the model was the same as in the group not following the model. The difference between the two groups was in the k_e_ values. In the group not following the model the k_e_ was higher than k_c_. The reason of this phenomenon is not known, but the distribution seems to be too complex. Nephrotoxicity can be excluded as a possible reason, because it decreases glomerular filtration and elimination rate [19]. The applied dose was approximately 10 mg/kg, lower than used earlier in rats [19] and this dose is much below the no observed adverse effect levels (NOAELs) of HPBCD in rats (50 mg/kg/day) [4,27]. Pharmacokinetic data revealed that FITC-HPBCD had fast elimination in mice, similar to HPBCD data in rats and humans. The t_1/2_ of FITC-HPBCD was between 6–8 min in the first 15 min and 9–20 min in the 30–60 or 45–60 min sampling period. Only two animals (belonging to the group following the model) had high t_1/2_, causing big deviation. The group not following the model had lower t_1/2_ in the last period. V_d_ values showed fast distribution, at 1 min these were between 4.7 and 7 mL, which were higher than the expected blood volume of mice. The two groups had similar V_d_ at 1 min of sampling, it was 5.55 ± 0.98 mL (*n* = 6) for the group following the model, and 5.85 ± 1.72 (*n* = 6) for the group not following the model (Table 1). It should be noted that the average V_d_ was 5.7 ± 1.34 mL (*n* = 12), which corresponds well with the extracellular fluid volume (5.8 ± 0.3 mL for 28.6 ± 1.2 g male mice) determined in wild-type C57BL6 mice [28]. According to these data, FITC-HPBCD rapidly distributes over the extracellular fluid after i.v. administration.

Measurement of the tissue distribution of FITC-HPBCD after 60 min of treatment showed that tissue accumulation could be detected only in kidneys. The major route of elimination of HPBCD is renal excretion and similar to earlier studies on rats with HPBCD, the target organs of FITC-HPBCD are kidneys in mice. Ex vivo fluorescence studies showed more details of distribution. After 30 min of treatment, significant fluorescence could be also detected in other organs like lung, liver and brain. It is in agreement with earlier findings, where the highest HPBCD concentrations were found in kidneys and lung of rats and in kidneys and liver of dogs [4]. Recently, we also found relatively higher ^68^Ga-NODAGA-HPBCD activity in the lung after 30 min compared to other organs [21]. Interestingly, we found FITC-HPBCD fluorescence in the brain after 30 min, which decreased to the control level at 60 min in accordance with the tissue distribution result. In experiments carried out with [^14^C]-HPBCD on wild type BALB/c (Npc1+/+) mice and Npc1−/− mice, it was found that the V_d_ at 2 min available to HPBCD exceeded the plasma and vascular volume of the brain, indicating a significant binding of HPBCD to the brain vasculature without crossing of the blood–brain barrier (BBB) [29]. The interaction of FITC-HPBCD and endothelial cells can explain the fluorescence of the brain, without BBB penetration. The molecular structure, hydrophilicity and high molecular weight predict that cyclodextrins do not penetrate biological membranes [30]. However, we recently showed that fluorescent cyclodextrins are able to enter Caco-2 intestinal epithelial cells by endocytosis [14,15]. To test the possible interactions of FITC-HPBCD with endothelial cells we treated HUVECs with this compound and investigated the cellular fluorescence. We found for the first time, that FITC-HPBCD localized in the cytoplasm of HUVECs in small endocytic vesicles after 30 min treatment. On the fluorescence microscopic images, numerous small vesicles can be observed in the cytoplasm of the treated cells. Interestingly, some autofluorescent vesicles can also be seen in the control cells. The autofluorescence of HUVECs was reported by other studies, but its origin is unknown [31]. The internalization and increase of cellular fluorescence were confirmed by flow cytometry. After 60 min incubation of FITC-HPBCD loaded cells with HBSS, the fluorescence was markedly decreased, but not completely. This indicates that endothelial cells can take part in the distribution of FITC-HPBCD in the body, but on the other hand, the endocytosis of FITC-HPBCD should be confirmed by other types of endothelial cells, like brain capillary endothelial cells.

Limitations of the study: The in vivo fluorescence imaging experiments were not suitable to detect the real-time organ distribution of FITC-HPBCD in mice. FITC-HPBCD in the highly perfused skin capillaries gives a strong fluorescence signal from the skin of the animals, obscuring the signal of the organs from the deeper layer. Ex vivo organ measurements were needed to reveal the organ distribution of FITC-HPBCD.

## 5. Conclusions

In conclusion FITC-HPBCD has similar pharmacokinetic properties to HPBCD, although we found some deviation from the applied two-compartment model. It should be noted that we tested this molecule on mice, while the available data originates from other species. The fast elimination, similar t_1/2_, V_d_ and tissue distribution show that FITC-HPBCD could be a suitable alternative model molecule to study and understand the pharmacological effect of HPBCD. The higher distribution in the lung can explain the pulmonary side effects of HPBCD, but on the other hand it may help to develop new targeting strategies. The great advantage of FITC-HPBCD is the easy, simple and sensitive detection, compared to HPBCD. The applied fluorescent technology can also help to test new cyclodextrin derivatives for targeted therapy. In addition, further studies are needed to test other administration routes.

## Figures and Tables

**Table 1:** Pharmacokinetic parameters of 6-deoxy-6-[(5/6)-fluoresceinylthioureido]-(2-hydroxypropyl)-β-cyclodextrin (FITC-HPBCD), administered intravenously to mice.

	Group F	Group NF		Group F	Group NF
Dose (µg)	250	250	Dose (µg)	250	250
t_1/2 (1–15 min)_	7.82 ± 1.48	6.13 ± 0.78	t_1/2 (1–15 min)_	7.5 ± 1.13	6.50 ± 0.31
t_1/2 (15–30 min)_	7.54 ± 0.52	35.01 ± 10.15	t_1/2 (15–45 min)_	13.55 ± 1.45	13.36 ± 3.05
t_1/2 (30–60 min)_	122.3 ± 109.4	16.61 ± 7.12	t_1/2 (45–60 min)_	20.06 ± 3.89	8.99 ± 4.54
k_e (1–15 min)_	0.092 ± 0.023	0.115 ± 0.017	k_e (1–15 min)_	0.095 ± 0.017	0.107 ± 0.006
k_e (15–30 min)_	0.092 ± 0.007	0.022 ± 0.009	k_e (15–45 min)_	0.052 ± 0.007	0.054 ± 0.013
k_e (30–60 min)_	0.022 ± 0.029	0.049 ± 0.020	k_e (45–60 min)_	0.036 ± 0.008	0.095 ± 0.044
k_c (1–15 min)_	0.109 ± 0.026	nd	k_c (1–15 min)_	0.142 ± 0.033	nd
V_d_ (mL)	4.86 ± 0.21	4.70 ± 1.27	V_d_ (mL)	6.25 ± 0.77	7.01 ± 0.81
CL (mL/min)	0.11 ± 0.15	0.25 ± 0.15	CL (mL/min)	0.23 ± 0.08	0.69 ± 0.38

Group F: pharmacokinetic parameters followed the two-compartment, first-order kinetic model. Group NF: pharmacokinetic parameters did not follow the two-compartment, first-order kinetic model. V_d_: Volume of distribution at 1 min. CL: clearance. nd: k_c_ values in Group NF could not be determined.

## Supplementary Materials

The following are available online https://www.mdpi.com/2218-273X/9/10/509/s1. Figure S1: Ex vivo fluorescence of the lung, 60 min after the i.v. administration of 0.5 mg FITC-HPBCD. Figure S2: In vivo fluorescence and X-ray imaging of FITC-HPBCD treated mice.
