# Supplementary figures and images for: Pharmacokinetic Properties of Fluorescently Labelled Hydroxypropyl-Beta-Cyclodextrin
Source: Biomolecules. 2019 Sep 20;9(10):509. doi: 10.3390/biom9100509 (PMC6843445; doi:10.3390/biom9100509)

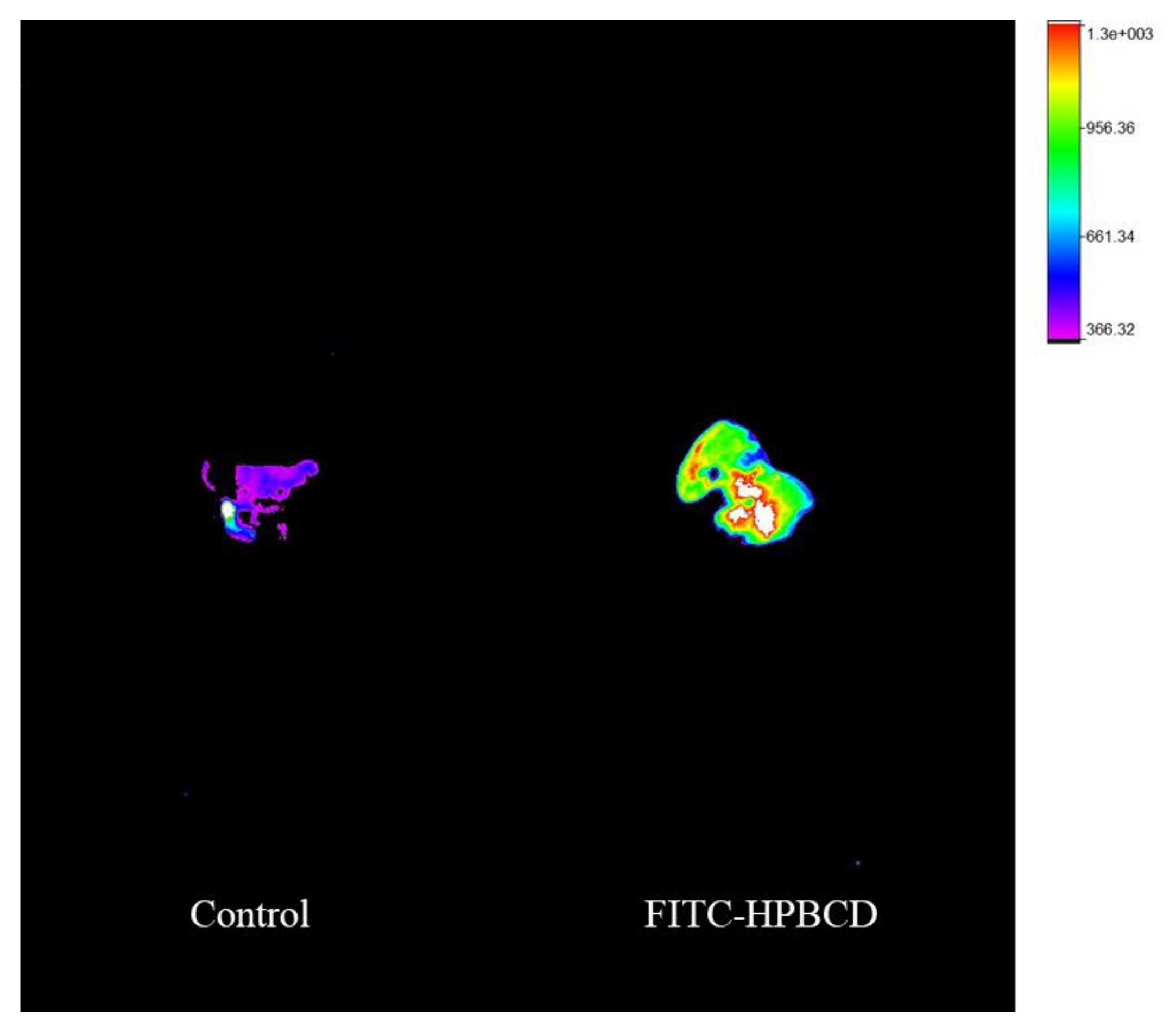

Supplement: Supplementary file 1 [file biomolecules-09-00509-s001.zip › Supplementary/SupplFig1.tif]

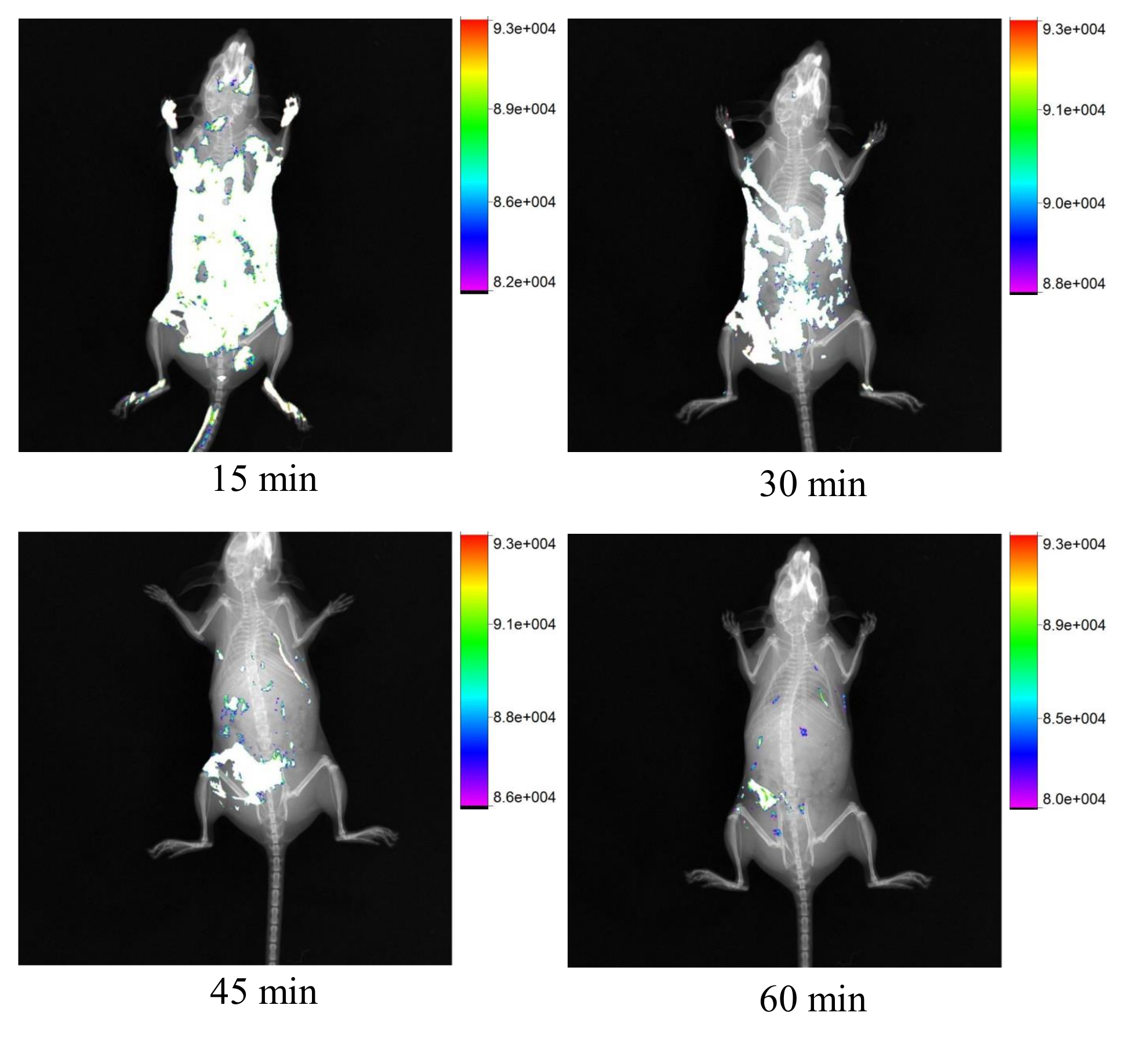

Supplement: Supplementary file 1 [file biomolecules-09-00509-s001.zip › Supplementary/SupplFig2.tif]
